# A brief review: history to understand fundamentals of electrocardiography

**DOI:** 10.3402/jchimp.v2i1.14383

**Published:** 2012-04-30

**Authors:** Majd AlGhatrif, Joseph Lindsay

**Affiliations:** 1 Department of Medicine, Medstar Union Memorial Hospital, Baltimore, MD, USA; 2 Department of Medicine, Medstar Washington Hospital Center, Washington, DC, USA

**Keywords:** history, electrocardiogram, electrocardiography

## Abstract

The last decade of the 19th century witnessed the rise of a new era in which physicians used technology along with classical history taking and physical examination for the diagnosis of heart disease. The introduction of chest x-rays and the electrocardiograph (electrocardiogram) provided objective information about the structure and function of the heart. In the first half of the 20th century, a number of innovative individuals set in motion a fascinating sequence of discoveries and inventions that led to the 12-lead electrocardiogram, as we know it now. Electrocardiography, nowadays, is an essential part of the initial evaluation for patients presenting with cardiac complaints. As a first line diagnostic tool, health care providers at different levels of training and expertise frequently find it imperative to interpret electrocardiograms. It is likely that an understanding of the electrical basis of electrocardiograms would reduce the likelihood of error. An understanding of the disorders behind electrocardiographic phenomena could reduce the need for memorizing what may seem to be an endless list of patterns. In this article, we will review the important steps in the evolution of electrocardiogram. As is the case in most human endeavors, an understanding of history enables one to deal effectively with the present.

The last decade of the 19th century witnessed the rise of a new era in which physicians used technology along with classical history taking and physical examination for the diagnosis of heart disease. The introduction of chest x-rays in 1895 and the electrocardiograph (electrocardiogram) in 1902 provided objective information about the structure and function of the heart (1). The original electrocardiograph employed a string galvanometer to record the potential deference between the extremities resulting from the heart's electrical activation. In the first half of the 20th century, a number of innovative individuals set in motion a fascinating sequence of discoveries and inventions that led to the 12-lead electrocardiogram as we know it now (2).

Electrocardiography today is an essential part of the initial evaluation for patients presenting with cardiac complaints. Specifically, it plays an important role as a non-invasive, cost-effective tool to evaluate arrhythmias and ischemic heart disease (2). As a first line diagnostic tool, health care providers at different levels of training and expertise frequently find it imperative to have the ability to interpret electrocardiograms; however, a high rate of misinterpretation has been noted among non-specialized physicians especially among trainees (3). It is likely that an understanding of the electrical basis of electrocardiograms would reduce the likelihood of error. An understanding of the disorders behind electrocardiographic phenomena could reduce the need for memorizing what may seem to be an endless list of patterns.

In this article, we will review the important steps in the evolution of electrocardiogram. As is the case in most human endeavor, an understanding of history enables one to deal effectively with the present. We believe that, by reviewing the historical underpinnings of electrocardiography, we may assist in their interpretation and in understanding the relationship between cardiac pathology and its electrocardiographic presentation (Fig. 1).

**Fig. 1:**
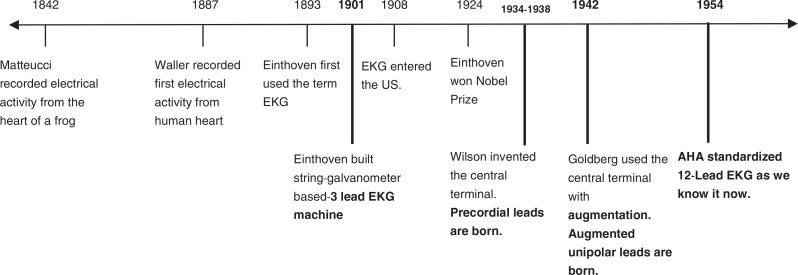
Timeline of clinically pertinent landmarks in the development of EKG, acknowledging the important efforts of other scientists in this process.

## Precursors of the electrocardiogram

**Fig. 2:**
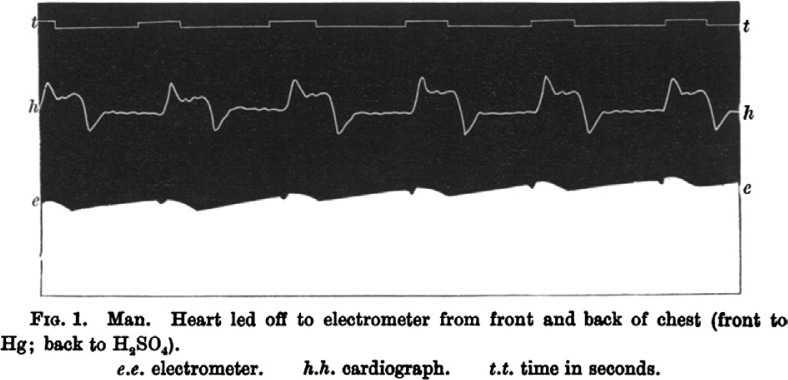
First human electrocardiogram recorded by Augustus D. Waller of St Mary's Medical School showing simultaneous electrometer and cardiograph tracings showing an electrical activity preceding every heart beat. From Ref. (6).

In the 1786, Dr. Luigi Galvani, an Italian physician and physicist at the University of Bologna, first noted that electrical current could be recorded from skeletal muscles. He recorded electrical activity from dissected muscles (4). In 1842, Dr. Carlo Matteucci, a professor of physics at the University of Pisa, demonstrated that electrical current accompanies every heart beat in a frog (5). Thirty-five years later, Augustus Waller, a British physiologist of St Mary's Medical School in London, published the first human electrocardiogram using a capillary electrometer and electrodes placed on the chest and back of a human. He demonstrated that electrical activity preceded ventricular contraction (Fig. 2) (6). In 1891, William Bayliss and Edward Starling, British physiologists of University College London, demonstrated triphasic cardiac electrical activity in each beat using an improved capillary electrometer (2, 7).

## Einthoven and the birth of clinical electrocardiogram

**Fig. 3:**
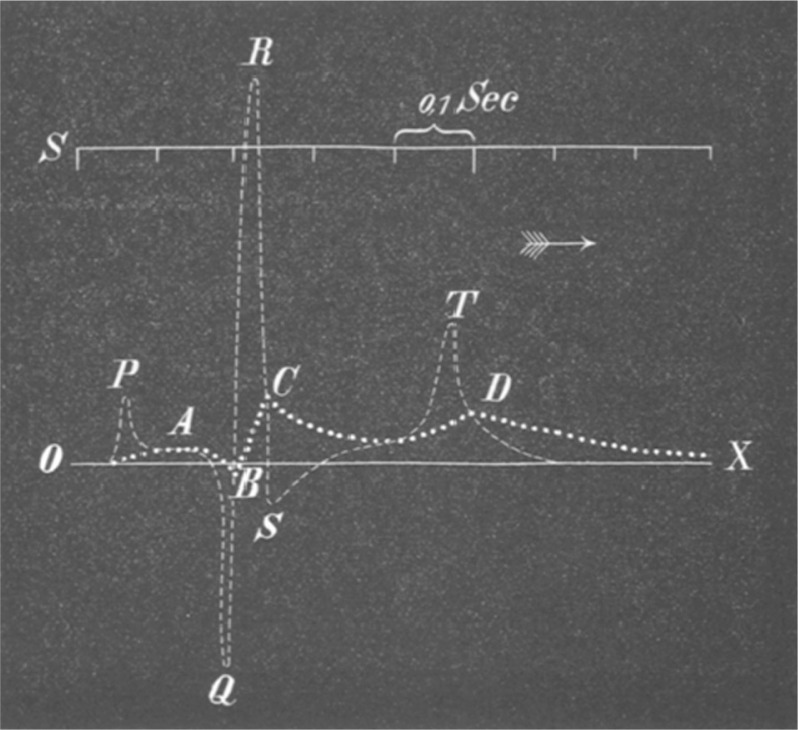
Two superimposed ECGs are shown. Uncorrected curve is labeled ABCD. This tracing was made with refined Lippmann capillary electrometer. The other curve was mathematically corrected by Einthoven to allow for inertia and friction in the capillary tube. He chose the letters PQRST for the corrected curve based on mathematical tradition of labeling successive point on a curve. From Ref. (8).

**Fig. 4:**
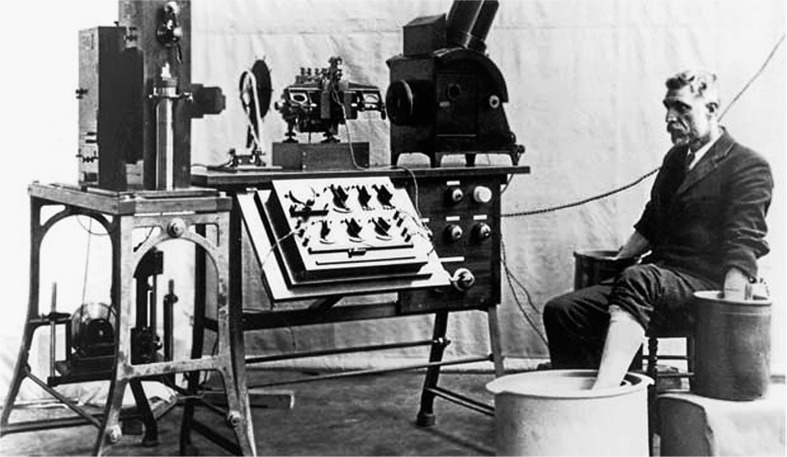
Old string galvanometer electrocardiograph showing the big machine with the patient rinsing his extremities in the cylindrical electrodes filled with electrolyte solution.

Dr. Willem Einthoven, a Dutch physiologist inspired by the work of Waller, refined the capillary electrometer even further and was able to demonstrate five deflections which he named ABCDE (Fig. 3) (8). To adjust for inertia in the capillary system, he implemented a mathematical correction, which resulted in the curves that we see today. Following the mathematical tradition established by Descartes (9), he used the terminal part of alphabet series (PQRST) to name these deflections. The term ‘electrocardiogram’ used to describe these wave forms was first coined by Einthoven at the Dutch Medical Meeting of 1893 (8, 10). In 1901, he successfully developed a new string galvanometer with very high sensitivity, which he used in his electrocardiograph. His device weighed 600 pounds (Fig. 4) (7, 11).

## Electrocardiogram from the bench to bedside

As the string galvanometer electrocardiograph became available for clinical use, improvements were made to make it more practical. Earlier electrocardiograms recorded by Waller used five electrodes, one on each of the four extremities and the mouth, with 10 leads derived from the different combinations (12). Einthoven was able to reduce the number of electrodes to three by excluding those which he thought provided the lowest yield, the right leg and the mouth electrodes. The resulting three leads were used to construct Einthoven's triangle, an important concept to this day (13). In 1924, Einthoven was awarded the Nobel Prize in physiology and medicine for the invention of electrocardiograph.

The first electrodes were cylinders of electrolyte solution in which extremities were rinsed (Fig. 4) (14). The positive leads were placed on the left arm and leg to produce positive deflections on electrocardiogram tracing as the normal electrical activation of the heart was noted to be from the right-upper quadrant to left-lower quadrant (14).

Sir Edward Schafer of the University of Edinburgh was the first to buy a string galvanometer electrograph for clinical use in 1908, and the first electrocardiogram machine was introduced to the United States in 1909 by Dr. Alfred Cohn at Mt. Sinai Hospital, New York (7).

## Three-lead electrocardiogram in clinical practice

During the first three decades of the 20th century, the three-lead electrocardiogram usage expanded especially after improvements were made to make it more portable (15). Electrocardiograms were initially used to study arrhythmias. In 1909, Sir Thomas Lewis of University College Hospital, London, discovered that ‘Delirium Cordis, ’ a clinical diagnosis of irregular heartbeat, was a result of atrial fibrillation using the electrocardiogram (16). After the recognition of myocardial infarction as a clinical entity in 1910, attempts were made to recognize electrocardiogram patterns suggestive of ischemic heart disease. By 1930, the importance of electrocardiogram in differentiating cardiac from non-cardiac chest pain was well recognized; in fact, some patterns were considered so characteristic that the electrocardiogram alone could be used to confirm the diagnosis of myocardial infarction (17).

## Precordial leads, the horizontal plane of the heart

**Fig. 5:**
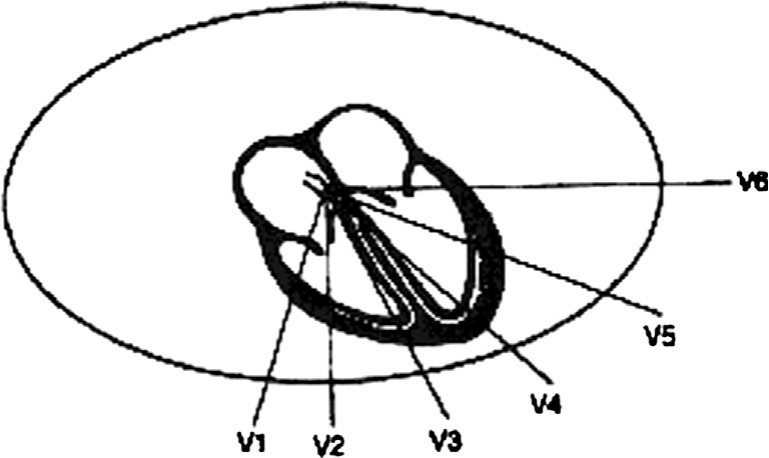
Wilson's precordial leads.

While the three-lead electrocardiogram was a satisfactory method to assess arrhythmias, it was soon recognized that there were ‘silent areas’ in the heart where, a myocardial infarction might not be detected (18). In 1934, Dr. Frank N. Wilson of the University of Michigan developed the concept of the ‘central terminal’. By connecting the three limb electrodes, a central negative lead reflecting a ‘ground’ or reference terminal was created. An electrode from the body surface connected through a galvanometer to this ground measured the potential difference between that point on the body and what can be thought of as zero. These ‘unipolar’ leads were in contrast to the ‘bipolar’ leads that measure the potential difference between two sites on the body surface. The unipolar lead could have theoretically been placed at any point on the body and, as a consequence, was termed an exploring lead. In 1938, the American Heart Association and the Cardiac Society of Great Britain published their recommendation for recording the exploring lead from six sites named V1 through V6 across the precordium. Thus, the chest leads were born (Fig. 5).

## Augmented unipolar leads

**Fig. 6:**
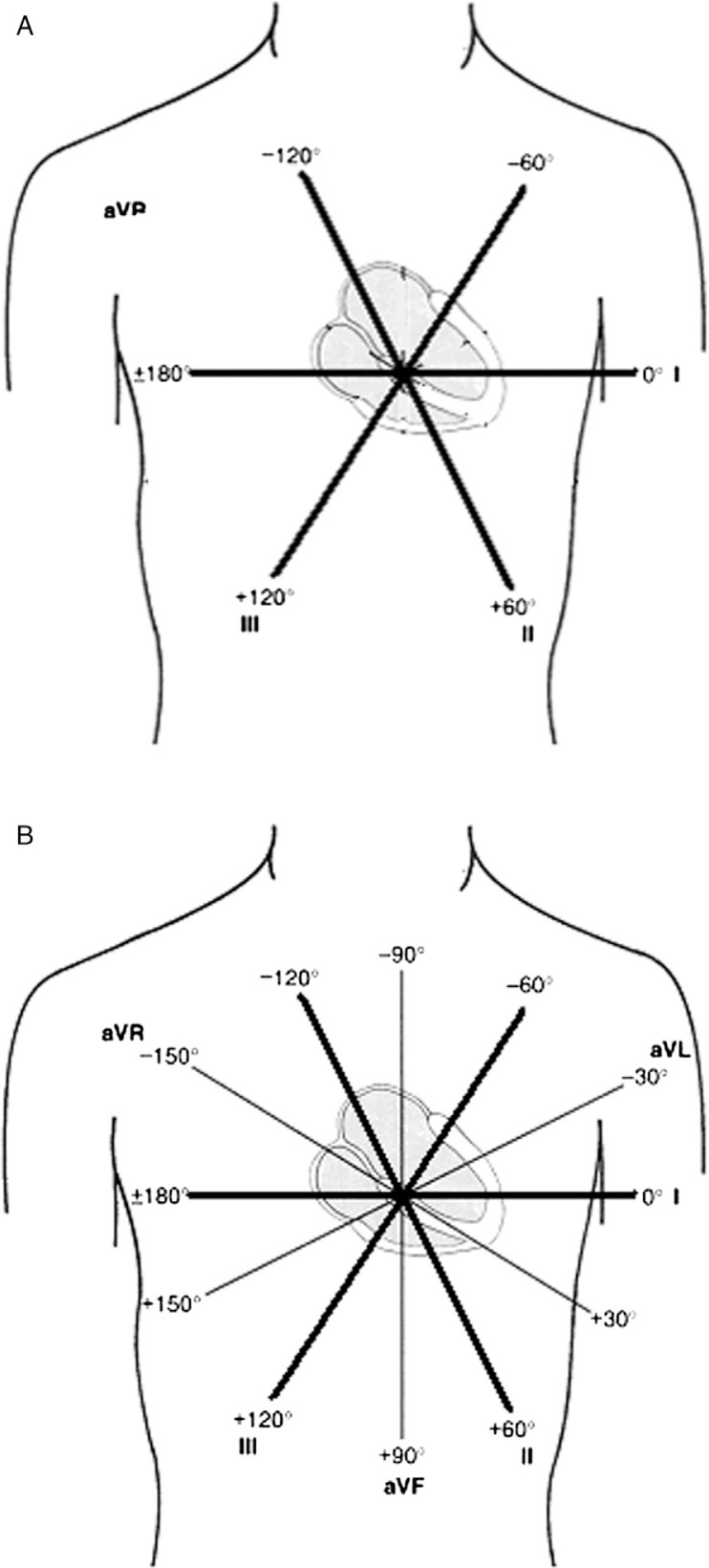
(A) Bipolar limb leads covering the frontal plane in 60° intervals. (B) The addition of the unipolar leads provided a more detailed coverage with 30° intervals.

Since the three-lead electrocardiogram covered the frontal plane with 60° increments (Fig. 6A), it seemed possible that there were uncovered segments potentially increasing the possibility of electrically silent myocardial pathology. In 1942, Dr. Emanuel Goldberger of Lincoln Hospital, New York, using Wilson's central terminal, constructed unipolar leads with the central (zero) terminal and connected to additional positive unipolar leads on each of the left and right arms and the left leg (Fig. 6B) (19). This method provided more detailed coverage of the frontal plane with 30° increments. Since the signal of these unipolar leads was small, Goldberger designed a method to augment these signals resulting on what we know now as the augmented unipolar limb leads a-VL, a-VR, and a-VF. Of note, this is the first time that a positive electrode was placed over the right arm, against the direction of the electrical activation, leading to the strange looking a-VR. The invention of the unipolar leads concluded the major advancement toward the 12-lead electrocardiogram as we know it now. In 1954, the American Heart Association published their recommendation for standardization of 12-lead electrocardiogram (20).

## Summary

Since Einthoven's original electrocardiogram, half a century passed until it evolved into the 12-lead electrocardiogram as we know it now. In each step in this seemingly slow process, physicians embraced the electrocardiogram as an essential clinical instrument; however, with time, they recognized the deficiencies in those earlier limited versions. It was this recognition of deficiency that pushed physicians and scientists to improve this technology allowing the optimization of this non-invasive tool.

Electrocardiography has played an important role in our understanding of heart disease. It together with its offspring, electrophysiology, remain the final arbiter of the nature of rhythm disturbances. Moreover, it retains great value in managing patients with ischemic heart disease. It was among the first bits of technology to supplement physicians’ clinical skills by providing objective data on the function and structure of the human body. Many researchers contributed to the development and refinement of electrocardiograph. We suggest that knowledge of the evolution of this most frequently used technology will assist in its interpretation.
